# Resistin serum levels and its association with clinical profile and carotid intima-media thickness in psoriasis: a cross-sectional study^☆^

**DOI:** 10.1016/j.abd.2022.10.011

**Published:** 2023-06-22

**Authors:** Sofia Makishi Schlenker, Sofia Inez Munhoz, André Rochinski Busanello, Matheus Guedes Sanches, Barbara Stadler Kahlow, Renato Nisihara, Thelma Larocca Skare

**Affiliations:** a Medicine Course, Faculdade Evangélica Mackenzie do Paraná, Curitiba, PR, Brazil; b Rheumatology Service, Hospital Universitário Evangélico Mackenzie, Curitiba, PR, Brazil

**Keywords:** Atherosclerosis, Arthritis, Psoriatic, Psoriasis, Resistin

## Abstract

**Background:**

Psoriasis is a protean disease associated with several comorbidities that may have increased levels of adiponectin such as resistin. This may affect the patients atherosclerotic risk.

**Objective:**

To study resistin levels in a sample of Brazilian patients with psoriasis and its association with clinical profile, comorbidities, and carotid Intima-Media Thickness (cIMT).

**Methods:**

This is a cross-sectional study of 119 individuals: 34 healthy controls and 85 patients with psoriasis, 42 of which with skin involvement only and 43 with psoriatic arthritis. Clinical and epidemiological data, measurement of PASI (Psoriasis Area Severity Index) and DAPSA (Disease Activity in Psoriatic Arthritis), lipid profile, cIMT by ultrasound were collected from medical records. Resistin serum levels were measured by ELISA.

**Results:**

Patients with psoriasis had higher resistin levels (p = 0.009) and worse cIMT (p = 0.0002) than controls. In the psoriasis sample, no associations of resistin levels with epidemiological, clinical findings, and activity indexes were found. Resistin serum levels were associated with the presence of diabetes (p = 0.008) and metabolic syndrome (p = 0.01) and correlated with total cholesterol (*r* = 0.26) and triglycerides (*r* = 0.33) but not with cIMT.

**Study limitations:**

This work is limited by its transversal design and by the limited number of patients included.

**Conclusion:**

Resistin serum levels are elevated in psoriasis patients. In this sample, clinical, epidemiological, and activity indexes were not linked to resistin serum levels, but atherosclerotic risk factors were.

## Introduction

Resistin is an adipocytokine with pleiotropic biological functions.^1^ It has been initially studied in mice, in which it has a link with obesity and diabetes^1^, ^2^, ^3^, ^4^; because of its action mediating insulin resistance, it was named resistin.^5^ In humans, it is expressed mainly in leukocytes and macrophages and stimulates immune cells to promote the secretion of proinflammatory cytokines such as TNF alpha, IL (Interleukin)-1B, and IL-6 through the nuclear factor KB signal pathway.^6^ It is connected to endothelial dysfunction. Obesity, atherosclerosis, cardiovascular events, and autoimmune diseases have been associated with elevation in serum resistin levels.^6^, ^7^

Psoriasis is a chronic inflammatory skin illness associated with obesity, insulin resistance, and cardiometabolic diseases^8^, ^9^, ^10^ in which resistin may play an active role. However, the studies on this association have held contradictory results. A meta-analysis including nine case-control studies with Caucasian and Asian patients suggested that resistin levels were markedly higher in psoriasis patients than controls.^11^ Some authors have found the association of resistin concentration with disease activity (measured by PASI or psoriasis area severity index) while others did not.^8^, ^12^ Nails involvement is another clinical aspect linked to resistin levels.^12^

Skin psoriasis is a protean disease with several clinical variants; the most common is psoriasis vulgaris or plaque psoriasis.^13^ It can also affect the nails and scalp and, in nearly 30% of the cases, progress to psoriatic arthritis that may assume several patterns affecting nearly any joint in the body.^13^ Moreover, the rate of association with comorbidities such as obesity, diabetes, hypertension, etc. may contribute to the heterogeneity of the studied population. This variety may be further amplified by predisposing genetic factors that change according to the geographic region of the studied population.^9^ So, the results found on resistin levels may suffer the influence of this diversity and this may explain some of the discrepancies found.

Herein, the authors studied a sample of Brazilian psoriasis patients aiming to compare resistin levels in psoriasis patients and controls and to study if these levels differ in psoriasis patients according to the presented clinical spectrum, disease activity, and associated cardiovascular risk factors.

## Material and methods

This is a cross-sectional study approved by the local Committee of Ethics in Research under protocol number 4.166.908. All participants signed consent. To be included psoriasis patients should have a psoriasis diagnosis done by a certified dermatologist or biopsy-proven and be older than 18 years of age. Patients were recruited from dermatology and rheumatology outpatient clinics from a single tertiary health care center from April 20 to December 21 and included according to appointment order and willingness to participate in the study. Individuals with any other associated chronic inflammatory disorders, chronic renal failure or pregnancy were excluded.

Data collection included:

a) Epidemiological data (age, sex, auto-declared ethnic background, smoking habits, alcohol use, exercise practice (at least 30 minutes 3 times/week).
b) Clinical data:
  On psoriasis: psoriasis type, PASI (psoriasis area and severity index)^14^ score, nail, and scalp involvement and used treatment;
  On psoriatic arthritis: articular involvement subtype, presence of dactylitis, uveitis, disease activity measured by DAPSA (or Disease Activity in Psoriatic Arthritis)^15^ and used treatment. PASI was calculated taking into account skin involvement severity (erythema, induration and desquamation) and percentage of the affected area. This index goes from 0 to 72 with higher values meaning worse disease.^14^ DAPSA is a psoriatic arthritis measurement of activity that takes into account the number of tender and swollen joints, patients’ perception of disease activity, pain, and C reactive protein levels. Values ≤ 4 mean arthritis remission; > 4 and ≤ 14, low disease activity; > 14 and ≤ 28, moderated activity and > 28, high disease activity^15^;
  On cardiovascular risk factors: Measurement of height and weight for BMI (body mass index), abdominal circumference and blood pressure; data on co-existence of diabetes, dyslipidemia, and hypertension. Patients were classified as having or not having metabolic syndrome according to NCEP-ATP III criteria.^16^
c) Biochemical data: Levels of total cholesterol, HDL cholesterol or high-density lipoprotein, LDL cholesterol or low-density lipoprotein, triglycerides, fasting glycemia, hemoglobin A1C, Cell Blood Count (CBC), ESR or erythrocyte sedimentation rate and CRP or C reactive protein were measured.
d) Measurement of carotid media thickness: Measurement of carotid Intima-Media Thickness (cIMT) was performed by a single investigator, blind for clinical data, with Esaote® ultrasound apparatus, high resolution, model MyLab40, in B-mode and with a linear transducer of 18 MHz. The patients were studied in a quiet, air-conditioned environment at 22 °C, in the supine position with the neck extended and rotated 45 degrees contralateral to the examined side. The carotid artery was observed in transverse and longitudinal planes, with measurement carried out at a distance of 10 to 20 mm of the carotid bifurcation, in the distal vessel wall 9.^17^ The examination was performed on both sides; for statistical purposes, the highest value was considered. The reference values used were 0.4 to 0.7 mm as normal cIMT; 0.8 to 1.4 mm as thickened cIMT (subclinical atherosclerosis); values greater than or equal to 1.5 mm, as atheroma.^18^
e) Resistin measurement: 5 mL of venous blood was collected from the peripheral vein and the serum was aliquoted and preserved in a −80 °C freezer until dosage. The measurement was done using a commercial ELISA kit (Elabscience, USA), detection range: 0.31∼2 ng/mL.

Epidemiological data, clinical data, blood collection and measurement of IMT were done simultaneously.

As a control, patients’ companions, paired for sex and age and without any chronic inflammatory disease were included.

### Statistical analysis

The distribution of obtained numerical data was studied by the Shapiro-Wilk test and their comparison through unpaired *t*-test or the Mann-Whitney test. Nominal data were compared using Fisher and the Chi-Squared test. Correlation studies of resistin levels with studied numerical variables (age, lipid and glycemic profile, ESR, CRP, PASI, DAPSA, BMI and cIMT) were done by Spearman test. The adopted significance was 0.05. The studies were done with the help of the MedCalc® Statistical Software version 20.015 (MedCalc Software Ltd, Ostend, Belgium; https://www.medcalc.org; 2021).

## Results

### Description of studied sample and comparison of psoriasis patients with controls

About 119 individuals were included: 34 controls and 85 with psoriasis patients, 42 (49.4%) with skin involvement only, and 43 (50.6%) with associated psoriatic arthritis. The description of the studied psoriasis sample is in Table 1.

**Table 1:** Clinical and demographical data of studied psoriasis sample

**Sex female/male – n (%)**	42 (49.4%) / 43 (50.5%)
**Age (years) ‒ mean ± SD**	51.6 ± 11.9
**Psoriasis skin subtypes**^a^	
Plaque psoriasis – n (%)	71/85 ‒ 83.5%
Palmoplantar	13/85 ‒ 15.2%
Pustular	2/85 ‒ 2.3%
Erythrodermic	2/85 ‒ 2.3%
**Nail involvement n (%)**	38/85 ‒ 44.7%
**Scalp involvement n (%)**	57/85 ‒ 67.0%
**PASI- median (IQR)**	2.9 (0.6‒6.6)
**Arthritis**^b^	43/85 ‒ 50.5%
Polyarticular – n (%)	18/43 ‒ 41.8%
Oligoarticular – n (%)	20/43 ‒ 46.5%
Distal interphalangeal – n (%)	5/43 ‒ 11.6%
Axial involvement – n (%)	14/43 ‒ 32.5%
DAPSA – median (IQR)	14.00 (4.27‒22.16)
**Systemic treatment**	
Methotrexate	24/85 ‒ 28.2%
Acitretin	5/85 ‒ 5.8%
Leflunomide	3/85 ‒ 3.5%
Anti TNF-alpha	21/85 ‒ 24.7%
Anti IL-17	10/85 ‒ 11.7%
Anti IL-12/23	9/85 ‒ 10.5%

n, number; IQR, Interquartile Range; PASI, Psoriasis Area and Severity Index; DAPSA, Disease Activity in Psoriatic Arthritis.

a 3 patients with combined forms.

b 14 patients with combined forms.

Table 2 shows the comparison of psoriasis patients and controls. This table shows the pairing data for age, sex and ethnic background and that smoking was more common among psoriasis patients. Psoriasis patients had higher body mass index, more diabetes mellitus, arterial hypertension, and metabolic syndrome than controls.

**Table 2:** Comparison of psoriasis patients with controls

	Psoriasis (n = 85)	Controls (n = 34)	p-value
**Epidemiological data**			
Mean age (years) (±SD)	51.6 ± 11.93	47.4 ± 14.3	0.11
Female/male sex	42/43	34/11	0.10
Autodeclared ethnic background			0.59
Caucasians	69 (81.1%)	29 (85.2%)	
Afro-descendants	16 (18.8%)	5 (14.7%)	
Smoking (n)			0.002
Current	22	2	
Ex-smokers	17	2	
No	46	30	
Exercise practice (n)	33 (38.8%)	12 (35.2%)	0.83
Alcohol use (n)	29 (34.1%)	13 (38.2%)	0.70

**Cardiovascular risk factors**			
Median Body Mass index – kg/m^2^ (IQR)	28.9 (26.0‒32.3)	26.4 (22.3‒31.1)	0.05
Arterial hypertension (n)	41 (48.2%)	7 (20.5%)	0.005^a^
Diabete mellitus (n)	22 (25.8%)	2 (5.8%)	0.01^b^
Dyslipidemia (n)	42 (49.4%)	10 (29.4%)	0.10
Abdominal circumference (cm) (IQR)	102 (96.0‒111.8)	94 (84.5‒107.0)	0.01
Median total cholesterol ‒ mg/dL (IQR)	193.0 (170.0‒225.0)	195.0 (157.0‒217.0)	0.49
Median HDL cholesterol ‒ mg/dL (IQR)	45.0 (40.0‒53.8)	48.9 (44.9‒56.2)	0.06
Median LDL cholesterol ‒ mg/dL (IQR)	121.4 (93.9‒144.7)	105.0 (87.8‒138.0)	0.15
Median triglycerides ‒ mg/dL (IQR)	136.0 (95.0‒200.5)	134.0 (102.0‒174.9)	0.58
Median fasting glucose ‒ mg/dL (IQR)	92.5 (85.0‒109.0)	90.5 (81.4‒95.9)	0.36
Median HbA1C (%) ‒ (IQR)	5.5 (5.3‒6.0)	5.6 (5.4‒5.9)	0.91
Metabolic syndrome (n)	33/84 (39.2%)	3/31 (9.6%)	0.002^c^

n, number; SD, Standard Deviation; IQR, Interquartile Range.

a OR = 3.5, (95% CI 1.4 to 8.9).

b OR = 5.5, (95% CI 1.4 to 25.0).

c OR = 6.0, (95% CI 1.8 to 19.8).

**Figure 1:**
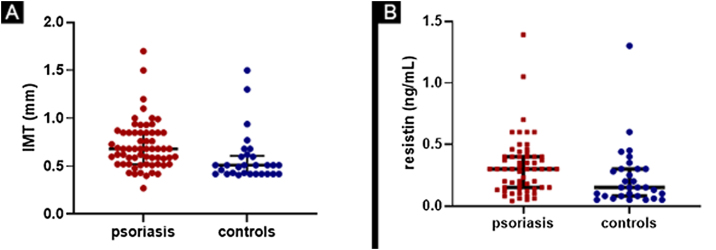
(A) Comparison of cIMT (carotid intima-media thickness) between psoriasis patients and controls. Patients with median value of 0.68 mm (0.52-0.85) and controls with median value of 0.51 mm (0.42-0.61), with p = 0,0002. (B) Comparison of resistin levels between psoriasis patients and controls. Patients with median value of 0.30 ng/mL (0.15-0.40) and controls with median value of 0.15 ng/mL (0.08-0.30) with p = 0.009

The comparison of results on cIMT and resistin serum levels between psoriasis patients and controls is in Fig. 1.

### Study of resistin serum levels according to psoriasis patients’ variables

Values of resistin serum levels according to epidemiological, clinical, and treatment variables are in Table 3 which shows that patients with metabolic syndrome and diabetes mellitus have higher serum levels of resistin.

**Table 3:** Resistin serum levels according to epidemiological, clinical and treatment variables in 85 psoriasis patients^a^.

	Resistin levels (ng/mL) in psoriasis patients with the variable	Resistin levels (ng/mL) in psoriasis patients without the variable	p-value
**Male sex**	0.30(0.17‒0.43)	0.30 (0.13‒0.40)	0.46
**Exposure to tobacco**	0.30 (0.15‒0.40)	0.30 (0.13‒0.40)	0.88
**Exercising**	0.20 (0.10‒0.35)	0.33 (0.15‒0.41)	0.08
**Plaque psoriasis**	0.30 (0.14‒0.40)	0.33 (0.22‒0.67)	0.16
**Palmo plantar psoriasis**	0.32 (0.14‒0.50)	0.30 (0.14‒0.40)	0.69
**Nail involvement**	0.30 (0.12‒0.407)	0.30 (0.16‒0.40)	0.61
**Scalp involvement**	0.30 (0.15‒0.70)	0.30 (0.10‒ 0.40)	0.65
**Articular involvement**	0.30 (0.15‒0.40)	0.30 (0.25‒0.40)	0.95
Polyarticular	0.30 (0.15‒0.40)	0.19 (0.13-0.45)	0,47
Oligoarticular	0.20 (0.13‒0.50)	0.30 (0.15-0.40)	0.93
Axial involvement	0.30 (0.13‒0.37)	0.30 90.14-0.40)	0.64
**Arterial hypertension**	0.325 (0.13‒0.47)	0.30 (0.15‒0.36)	0.23
**Diabetes mellitus**	0.40 (0.30‒0.50)	0.28 (0.13‒0.37)	0.008
**Dyslipidemia**	0.35 (0.14‒0.50)	0.21 (0.15‒0.35)	0.06
**Metabolic syndrome**	0.40 (0.20‒0.50)	0.25(0.13‒0.35)	0.01
**Methotrexate treatment**	0.30 (0.14‒0.50)	3.00 (0.14‒0.40)	0.55
**Anti TNF treatment**	0.30 (0.18‒0.44)	0.30 (0.14‒0.40)	0.61
**Anti IL-17 treatment**	0.28 (0.12‒0.37)	0.30 (0.15‒0.42)	0.30

a All measurements expressed in median values and interquartile range.

Table 4 displays the results of the correlation of serum levels of resistin with lipid and glycemic profile, ESR, CRP, PASI, DAPSA, age, BMI, and cIMT. Total cholesterol and triglycerides showed a modest positive correlation.

**Table 4:** Correlation studies of resistin serum levels with inflammatory markers, glycemic and lipid profile, age, anthropometric data, and carotid media-intima thickness

	Spearman *r*	95% CI	p-value
**Age**	0.22	−0.03 to 0.46	0.08
**PASI**	−0.04	−0.30 to 0.22	0.73
**DAPSA**	0,07	−0,34 to 0,46	0,72
**Erythrocyte sedimentation rate**	−0.22	−0.46 to 0.05	0.10
**C reactive protein**	0.05	−0.34 to 0.46	0.72
**Body mass index**	0.25	−0.01 to 0.48	0.05
**Abdominal circumference**	0.13	−0.13 to 0.38	0.32
**Total cholesterol**	0.26	0.003 to 0.49	0.04
**HDL cholesterol**	0.02	−0.24 to 0.28	0.86
**LDL cholesterol**	0.10	−0.16 to0.36	0.42
**Triglycerides**	0.33	0.07 to 0.55	0.01
**Glucose levels**	0.14	−0.13 to 0.39	0.29
**Hemoglobin A1C**	0.12	−0.15 to 0.38	0.37
**cIMT**	−0.12	−0.40 to 0.17	0.41

95% CI, Confidence Interval 95% PASI, Psoriasis Area and Severity Index; DAPSA, Disease Activity in Psoriatic Arthritis; HDL, High Density Lipoprotein; LDL, Low Density Lipoprotein; cIMT, Carotid Media-Intima Ihickness.

## Discussion

The results of the present study have confirmed the high association of psoriasis with the atherosclerotic risk factor. Indeed, these patients also had high values for cIMT showing the outcomes of such association. Resistin serum levels were higher in psoriasis than controls and this adipokine may be one of the actors underlying the found picture.

However, when the sample with psoriasis patients was studied, the observed values of resistin levels did not associate with the degree of skin involvement nor with the presence or disease activity in those with psoriatic arthritis. Nevertheless, it is important to observe that the psoriasis-studied patients in this sample had a low degree of skin involvement measured by PASI as well as the median inflammatory index in the case of arthritis was consistent with low disease activity. The patients from this sample were from a tertiary health care outpatient clinic that treats patients intensively aiming to achieve disease remission.

The study of resistin serum levels in association of PASI has detained contradictory results. Takahashi et al.,^8^ studying Japanese patients with psoriasis found a link between resistin levels and PASI scores and that resistin serum levels decreased with treatment. Coban et al.,^19^ studying psoriasis patients from Turkey and the association of PASI with several adipocytes prior to and after treatment, could not prove that serum resistin levels decreased with PASI improvement. Özdemir et al.^12^ also did not find any correlation between resistin and PASI but a positive correlation with NAPSI (Nail Psoriasis Severity Index). These discrepancies may be partially explained by the different disease spectrum of psoriatic patients as mentioned before, but also by the influence of adopted treatment regimens such as anti-TNF alpha therapy. Resistin not only stimulates the secretion of TNF alpha but also has its production induced by this same cytokine suggesting the existence of a pathogenic cycle that fosters inflammatory cytokine production, along with resistin secretion.^20^, ^21^ In the present work, resistin levels in those using anti-TNF therapy did not differ from those not using it.

Herein, variables linked to resistin levels were those of comorbidities such as dyslipidemia and diabetes. This association is not restricted to psoriasis being observed in other clinical situations such as in overweight and obese patients with type 1 DM,^22^ chronic kidney disease,^23^ coronary artery disease, and atherothrombotic stroke^24^ suggesting that expansion and metabolic dysregulation of white fat tissue associated to obesity leading to adipocytokine secretion and inflammation is the underlying link in such context. Macrophages infiltrating white adipose tissue found in visceral and subcutaneous fat are considered to be one of the main sources of resistin in humans.^25^ Psoriasis patients are more frequently obese than the general population as it was found in the present work, and obesity has been considered a risk factor for psoriasis appearance.^26^ Curiously, Toussirot et al.^26^ found that visceral fat accumulates more in patients with skin psoriasis only than in those with psoriasis and associated arthritis. The authors could not prove differences in resistin levels in the groups with and without arthritis.

One unexpected finding was the lack of correlation between resistin levels and cIMT, even though cIMT was higher in psoriasis patients than controls. One possible explanation is that resistin serum levels reflect the measurement of this molecule at a single point in time, while atherosclerosis is a process built over a period. Moreover, it is worthwhile to notice that the cIMT of psoriasis patients was not very high (the mean value was 0.68 mm and the upper value of 0.85 mm) probably due to a tight clinical surveillance; this could have had some influence on the results.

This work is limited by its transversal design and by the limited number of included patients. It is main value is to demonstrate that there is an association of resistin levels in psoriasis patients, mainly in those with comorbidities and that the comorbidities are, probably, the main target of treatment to avoid elevation of this serum adipocytokine in this setting.

## Conclusion

This study has shown that, in a sample of Brazilian patients with psoriasis, resistin serum levels are elevated and associated with the presence of DM and metabolic syndrome and correlate with serum triglycerides and total cholesterol. No correlation of resistin serum levels with disease activity or cIMT was found.

## Ethics approval

All procedures performed in studies involving human participants were in accordance with the ethical standards of the institutional research committee and with the 1964 Helsinki Declaration and its later amendments or comparable ethical standards. This study was approved by the Committee of Ethics in Research from Evangelic Mackenzie School of Medicine under protocol number 4.166.908

## Financial support

None declared.

## Authors' contributions

Sofia Makishi Schlenker: Approval of the final version of the manuscript; Design and planning of the study; Drafting and editing of the manuscript; Collection, analysis, and interpretation of data; Effective participation in research orientation; Intellectual participation in the propaedeutic and/or therapeutic conduct of the studied cases; Critical review of the literature; Critical review of the manuscript.

Sofia Inez Munhoz: Approval of the final version of the manuscript; Design and planning of the study; Drafting and editing of the manuscript; Collection, analysis, and interpretation of data; Effective participation in research orientation; Intellectual participation in the propaedeutic and/or therapeutic conduct of the studied cases; Critical review of the literature; Critical review of the manuscript.

André Rochinski Busanello: Approval of the final version of the manuscript; Collection, analysis, and interpretation of data; intellectual participation in the propaedeutic and/or therapeutic conduct of the studied cases; Critical review of the literature.

Matheus Guedes Sanches: Approval of the final version of the manuscript; Collection, analysis, and interpretation of data; Intellectual participation in the propaedeutic and/or therapeutic conduct of the studied cases; Critical review of the literature

Barbara Stadler Kahlow: Approval of the final version of the manuscript; Collection, analysis, and interpretation of data; Intellectual participation in the propaedeutic and/or therapeutic conduct of the studied cases; Critical review of the literature

Renato Nisihara: Statistical analysis; Approval of the final version of the manuscript; Drafting and editing of the manuscript; Collection, analysis, and interpretation of data; Intellectual participation in the propaedeutic and/or therapeutic conduct of the studied cases; Critical review of the literature; Critical review of the manuscript

Thelma Skare: Statistical analysis; Approval of the final version of the manuscript; Design and planning of the study; Drafting and editing of the manuscript; Collection, analysis, and interpretation of data; Effective participation in research orientation; Intellectual participation in the propaedeutic and/or therapeutic conduct of the studied cases; Critical review of the literature; Critical review of the manuscript

## Conflicts of interest

None declared.
